# Dense bidisperse suspensions under non-homogeneous shear

**DOI:** 10.1038/s41598-023-41587-3

**Published:** 2023-08-31

**Authors:** Alessandro Monti, Marco Edoardo Rosti

**Affiliations:** Complex Fluids and Flows Unit, Okinawa Institute of Science and Technology Graduate University, 1919-1 Tancha, Onna-son, Okinawa, 904-0495 Japan

**Keywords:** Engineering, Physics

## Abstract

We study the rheological behaviour of bidisperse suspensions in three dimensions under a non-uniform shear flow, made by the superimposition of a linear shear and a sinusoidal disturbance. Our results show that (i) only a streamwise disturbance in the shear-plane alters the suspension dynamics by substantially reducing the relative viscosity, (ii) with the amplitude of the disturbance determining a threshold value for the effect to kick-in and its wavenumber controlling the amount of reduction and which of the two phases is affected. We show that, (iii) the rheological changes are caused by the effective separation of the two phases, with the large or small particles layering in separate regions. We provide a physical explanation of the phase separation process and of the conditions necessary to trigger it. We test the results in the whole flow curve, and we show that the mechanism remains substantially unaltered, with the only difference being the nature of the interactions between particles modified by the phase separation.

## Introduction

Dense suspensions of hard particles immersed in a Newtonian solvent are common in many natural and industrial applications, such as the pharmaceutical industry (e.g. the process of blending powders for tablets production), food industry (e.g. powdery products), powder metallurgy (e.g. the processes of compacting and sintering blended pulverized metals), and sediment transport (e.g. transportation of nutrients and landscape shaping)^1–5^, with applications reaching biofluidics and bacterial suspensions^6^. These materials, when subject to a shear-rate $\dot{\gamma }$, often show peculiar rheological behaviours that can increase or decrease the fluidity of the suspension. Among these behaviours, shear-thickening, i.e. a reduction of the fluidity properties of the suspension when the latter is subject to an increasing $\dot{\gamma }$, is of particular interest. Shear thickening is perhaps the most astonishing and most studied non-Newtonian behaviour of dense suspensions, and until few years ago it was far from being understood. The reduction of the fluidity is now attributed to the modification of the internal microstructure of the fluid^7^; a recent theory experimentally proved that shear-thickening is triggered by the close interactions between the particles that appear in the form of frictional contacts (due to the microscopic asperities on the surface of the particles) and constrain their relative movements, causing an enhancement of the viscosity of the suspensions^8–18^. The intensity of the shear-thickening can be quantified as an increase of the effective viscosity of the suspension $\eta _r$ and becomes stronger as the volume fraction of the suspension $\phi$ increases, until it displays an abrupt enhancement, behaviour known as discontinuous shear thickening^19,20^. The dependence on $\dot{\gamma }$ and $\phi$ of the rheological properties generally characterizes the mechanics of the suspensions within the shear-thickening regime. When the particles are suspended in a simple homogeneous shear flow^21^, demonstrated that such dual dependence can be reduced to a single parameter in analogy to granular flows^22^. In fact, specifying the macroscopic friction coefficient $\mu =\sigma _{12}/\Pi$ uniquely sets the volume fraction and the shear-rate, and general constitutive laws $\eta _r=\eta _r(\mu )$ and $\phi =\phi (\mu )$ can be formulated^21,23^. In the previous relation, $\sigma _{12}$ is the shear component of the stress tensor $\sigma$ and $\Pi$ is the pressure, related to the trace of $\sigma$. This powerful general outcome, however, fails when dealing with spatial or temporal inhomogeneity within the flow. In fact, phenomena such as subyielding and overcompaction^24–26^, particles migration^27–29^ or flow instabilities that can lead to banding^30–33^ and segregative phenomena^34^ cannot be captured by such constitutive-law, since it would require a two-phase description^23,35^. The route towards a complete model able to describe real suspensions that involve the aforementioned phenomena requires more studies that shed some light onto the elusive physical mechanisms activated by the inhomogeneities.

In this direction, we propose a three-dimensional numerical study that aims at analysing non-trivial phenomena generated when bidisperse particles are suspended in an inhomogeneous Newtonian flow characterized by the linear super-imposition of a plain shear flow and a sinusoidal velocity profile, i.e.,  with zero mean but a non-uniform shear-rate. Similar numerical experiments were carried out on two-dimensional granular flows: e.g.,^36^ studied a Kolmogorov-like flow of soft athermal disks close to the jamming transition and found that stress profiles can be reproduced by nonlocal constitutive relations that account for fourth-order gradients (see also^37^).

The importance of studying non-uniform profiles is suggested e.g., by the experimental shear-rheometer, where the condition of plain shear is hard to reach due the presence of the solid walls that inevitably introduce local inhomogeneity. Also, the present configuration is a quite general configuration of any pressure driven flows in channels, which are characterised by a non-uniform mean shear-rate^38^. In particular, the goal of the work is to trigger a mechanism of particles migration that results in a demixing of the two phases. In this sense, many works dealt with dense granular flow, where demixing effects (e.g., segregation) are of paramount interest to control and improve the industrial products quality^34,39–46^. To unravel the physical mechanisms governing the segregation, many recent studies focused on the the single intruder particle limit^47–53^ since it provides a simple, yet effective, test-case to investigate the problem. In particular^48^, carried out 2D numerical simulations where they proposed a method to measure the force acting on the intruder. This method was based on a virtual spring attached to the intruder that constrained it through a restoring force to oscillate around an equilibrium position. In this way, the authors were able to measure the force acting on the intruder and to derive an expression that modelled the segregative force as a function of the local pressure and the local shear-stress gradients. Following this seminal work^50,51^, showed that the segregation force is insensitive to the shear-stress gradients when the granular flow is subject to a linear velocity profile (typical of free-surface flows). However, when the velocity profile is non-linear, a higher-order correction that takes into account the local shear-rate gradients has to be included to correctly model the segregative force^53^. While these results have a great potential outcome to reach a unified continuum model, the studies are actually limited to a single intruder particle, and the origin of the segregation is still unclear. Finally, these models are limited to granular flows, where only contacts forces acting between the particles are modelled. A few studies on dilute and semi-dense suspensions are available in the literature^54–56^, where the authors mainly focused on the diffusivity of the suspensions and on some aspects of the shear-induced segregation.

In this work, we tackle these open-questions via a three-dimensional numerical investigation that studies the rheology of a dense bidisperse suspension, with high dispersion ratio^57^, driven by a linear combination of a plain shear-flow and a sinusoidal disturbance. We will exploit the numerical techniques to study the effect of the amplitude and of the wavenumber of the disturbance and investigate possible layering effects through a parametric study. The reference case will be a dense suspension sheared with a uniform shear-rate in the shear-thickening regime. The rheological quantities will be compared to that case, with particular interest in the effective viscosity of the suspension. We will then study in detail the mechanisms that the inhomogeneity triggers, with a complete analysis of the stress tensor and the breakdown of its contribution. Finally, we will study the effect of such driving flow for the complete flow curve. To the Authors’ knowledge, this is the first three-dimensional complete study with this type of inhomogeneity. The novelty lies (i) in the numerical models adopted (e.g., the introduction of the frictional contacts, described in “Methodology” section), (ii) in the very large number of particles introduced for a numerical study, i.e. $N=2^{16}$, and (iii) in the full analysis of the stress tensor of the suspension to identify key contributions to the segregation.

## Methodology

We carried out three-dimensional numerical simulations of a dense suspension of rigid, spherical particles, not subject to Brownian motions, immersed in a Newtonian fluid with viscosity $\eta _0$. The suspension is driven by a sum of a plain shear-flow, $u_1 = \overline{\dot{\gamma }} x_2$, characterised by the shear-rate $\overline{\dot{\gamma }}$ (all the quantities $\overline{\cdot }$ within the manuscript refer to their mean value along the shearwise direction *y*, while the quantities $\cdot (y)$ to their local value) and a sinusoidal disturbance in the velocity of the form

1 $$\begin{aligned} u_i=A \overline{\dot{\gamma }}a_0 \sin {\left( n\kappa _0 x_j\right) }, \end{aligned}$$

where *A* is the amplitude of the disturbance, $a_0$ is the reference length-scale of the problem (typically the radius of the smallest particle of the suspension), $\kappa _0=2\pi /L$ is the fundamental wavenumber of the signal (being *L* the size of the computational domain), *n* an integer that sets the wavenumber of the signal and $x_j$ the direction of the wave. In particular, the subscripts *i* and *j* span the spatial three-directions in a Cartesian frame of reference, where $x_1$, $x_2$ and $x_3$ (sometimes also referred to as *x*, *y* and *z*) are adopted to identify the streamwise, shearwise and spanwise directions, and $u_1$, $u_2$ and $u_3$ to identify the corresponding velocity components (*u*, *v* and *w*). In Eq. (1), the constraint $i\ne j$ is imposed.

**Figure 1:**
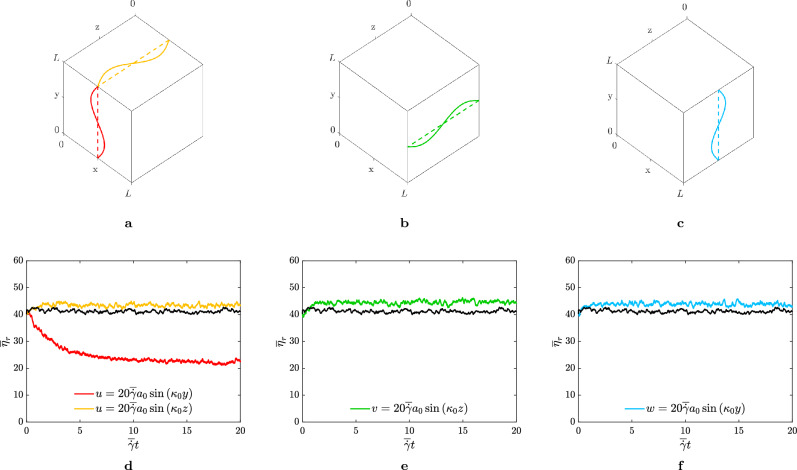
Top row, (**a**–**c**) sketch of the sinusoidal disturbance analysed in the respective columns. Bottom row, panels (**d**–**f**) time-history in terms of strain $\gamma$ of the relative viscosity $\overline{\eta }_r$. The colours distinguish the different cases, with the black solid lines referring to the values of $\overline{\eta }_r$ for the particles suspended in a plain shear-flow.

The suspensions considered are composed of $N=2^{16}$ particles and are binary, i.e. the particles have two different sizes, with radii $a_1=a_0$ and $a_2=3 a_0$. The two phases are dispersed with relative volume $V_2/V_1=0.25$. The computational box containing the particles is a cube with size designed to contain the desired volume fraction $\overline{\phi }=0.50$. The uniformity of the shear-flow at the edges of the domain is preserved through the adoption of the 3D-periodic Lees-Edwards boundary conditions^58^.

The numerical investigation is performed using a validated and publicly available software, *CFF-Ball-0x*^59^. The software tackles the translational and rotational dynamics of the particles by solving the Newton-Euler equations,

2 $$\begin{aligned} {\left\{ \begin{array}{ll} m_i \dfrac{\textrm{d}\varvec{u}_i}{\textrm{d}t} &{}= \sum \limits _{j=1}^{N_H} \varvec{F}^H_{ij} + \sum \limits _{j=1}^{N_C} \varvec{F}^C_{ij} + \sum \limits _{j=1}^{N_E} \varvec{F}^E_{ij}, \\ \mathbb {I}_i \dfrac{\textrm{d}\varvec{\omega }_i}{\textrm{d}t} + \varvec{\omega }_i \times (\mathbb {I}_i \varvec{\omega }_i) &{}= \sum \limits _{j=1}^{N_H} \varvec{T}^H_{ij} + \sum \limits _{j=1}^{N_C} \varvec{T}^C_{ij} + \sum \limits _{j=1}^{N_E} \varvec{T}^E_{ij}, \\ \end{array}\right. } \end{aligned}$$

where the subscript *i* indicates the particle $i\in [1,N]$, being *N* the number of particles. The right-hand side of System (2) lists the forces and torques that are applied to the centre of mass of the *i*th particle, with mass $m_i$ and inertia tensor $\mathbb {I}_i$ and cause a variation in the translational and angular velocities of the particles, here denoted by the symbols $\varvec{u}_i$ and $\varvec{\omega }_i$, respectively. Such forces and torques result from the particle-fluid and particle-particle interactions of the *i*th particle with its *j*th neighbour. The nature of the interactions is indicated by the superscripts *H*, *C* and *E*, i.e. hydrodynamics, inelastic contacts and electrochemical potentials, respectively.

Here, we give a brief overview of the models used to support the three aforementioned contributions. For hydrodynamics, we utilized the approach developed by^60^ and^11^, which considers the effects of a dense suspension of rigid particles in a low-Reynolds-number flow. These particles experience a Stokes drag and a pair-wise lubrication force^11^ that arises from the relative motion of nearby particles, leading to the squeezing of fluid between them. To account for these forces and torques, we employed a linear relationship between velocities (angular velocities) and forces (torques)^11,60^. This relationship is expressed as $\varvec{F}^H=-\mathbb {R}(\varvec{u}-\varvec{U}^\infty )$, where $\mathbb {R}$ is a resistance matrix that takes into account only the dominant near-field divergent elements resulting from the squeeze, shear, and pump modes, as described in the work by^11^. The far-field effects are neglected.

The stick-and-slide method^61^ is used to model the contact contribution, which simulates inelastic contacts with spring-dashpot systems that become active when two spheres overlap. The force is expressed as $\varvec{F}^C=(k_n\delta + \gamma _n\dot{\delta })\varvec{n} + k_t\xi \varvec{t}$, with the spring-dashpot systems oriented along the normal (centre-to-centre) $\varvec{n}$ and tangential $\varvec{t}$ directions, characterized by the spring and dashpot constants $(k_n$,$\gamma _n)$ and $(k_t$,$\gamma _t=0)$, respectively. The values $\delta$ and $\xi$ indicate the normal and tangential displacements of the springs, and $\dot{\delta }$ represents the normal projection of the overlapping velocity. The Coulomb’s law, $|\varvec{F}^C_t|\le \mu |\varvec{F}^C_n|$, applies to the tangential contribution, where $\mu$ is the frictional coefficient, set to $\mu = 0.5$ for all the scenarios examined in this work, as in our previous study^18^ where we compared our results with experimental data.

The last contribution considered in this work is the electrochemical properties of the suspension, modelled as a sum of an inter-particle, distance-decaying repulsion force and an attraction force in van der Waals form^16,62^. The resulting force is $\varvec{F}^E = \varvec{F}^R+\varvec{F}^A$, where the where the superscripts *E*, *R*, *A* stand for electrochemical, repulsive, and attractive, respectively. The repulsive contribution is given by $\varvec{F}^R=-F_0 e^{-d/L_s}\varvec{n}$, where $F_0$ is the magnitude of the force, $L_s$ is the screening length, and *d* is the particle-particle surface distance, while the attractive force can be written as $\varvec{F}^A = H_A\overline{a}/12(d^2+\varepsilon ^2) \varvec{n}$, where $H_A$ is the Hamaker constant, $\overline{a}$ is the harmonic mean radius of the two particles involved, and $\varepsilon$ is a smoothing term to eliminate the singularity when the two particles touch, i.e. $d = 0$.

The physics of the suspension described by the system of equations (2) is governed by several parameters. By analyzing the translational equation of (2) and combining the dimensional quantities, we can use Buckingham’s $\Pi$ theorem to identify a set of non-dimensional groups that can be adjusted to control the desired dynamics of the suspension. When using the hydrodynamic scales as the fundamental independent quantities, we obtain three non-dimensional groups. The first is the Stokes number,

3 $$\begin{aligned} St = \rho _p a_0^2 \dot{\gamma }/\eta _0, \end{aligned}$$

which compares the time-scale of the particles with that of the hydrodynamics and arises from the inertial term. In the equation above, $\rho _p$ is the density of the particles (equal to the density of the carrier fluid in the cases we considered), $a_0$ is the typical length scale of the system (the radius of the particles of the smallest phase of the suspension), $\eta _0$ is the viscosity of the carrier fluid and $\dot{\gamma }$ is the shear-rate applied to the suspension. The constraint $St \ll 1$ is applied to enforce the inertialess regime. The second number is the non-dimensional The second is the non-dimensional stiffness

4 $$\begin{aligned} \hat{k}=k_n/(\eta _0 a_0 \dot{\gamma }), \end{aligned}$$

which evaluates the importance of the contacts contributions relative to the hydrodynamic term. The constraint $\hat{k}\gg 1$ is imposed to ensure that the particles behave as rigid^11^. The normal spring constant $k_n$ is considered as the dominant term of the spring-dashpot systems, and additional constraints are applied to enforce this assumption. In particular, we impose $k_t=2/7k_n$ and $\gamma _n\dot{\gamma }/k_n \ll 1 \sim O(10^{-7})$, where the latter is the relaxation time of the spring-dashpot system. The third non-dimensional group is the equivalent shear-rate

5 $$\begin{aligned} \hat{\dot{\gamma }} = |\varvec{F}^E (d=0)|/(\eta _0 a_0^2 \dot{\gamma }) = \dot{\gamma }_0/\dot{\gamma }, \end{aligned}$$

which measures the time-scale introduced by the electrochemical contribution and can be modified to impose a shear-rate dependent rheology on the suspension. A table that recaps the parameters adopted in this work is reported in the Supplementary Material.

**Figure 2:**
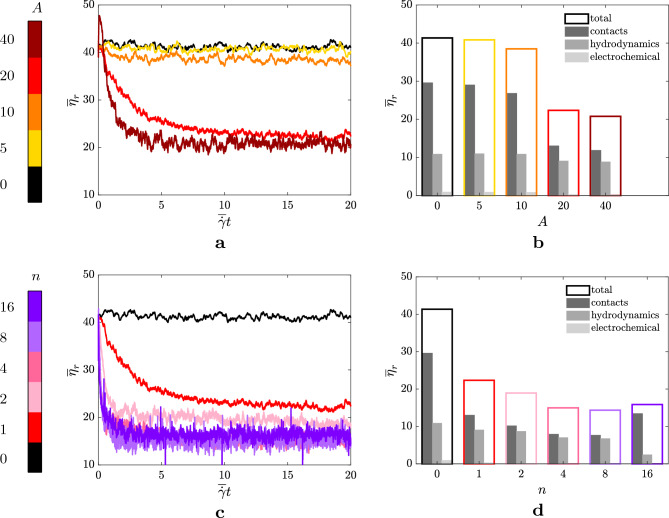
(**a**) and (**c**) time-history in terms of strain $\gamma$ of the relative viscosity $\eta _r$ for the disturbance $u=A \overline{\dot{\gamma }} a_0 \sin {\left( n \kappa _0 y \right) }$. (**b**) and (**d**) contributions to the mean relative viscosity $\overline{\eta }_r$ accumulated at statistically-steady state of the contacts, hydrodynamics and electrochemical potentials (the respective colours are shown in the legend within the panels). The panels in the top row, i.e. (**a**) and (**b**), show the trends of $\overline{\eta }_r$ varying the amplitude *A* of the disturbance with $n=1$. The panels in the bottom row, i.e. (**c**) and (**d**), show the trends of $\overline{\eta }_r$ as a function of the wavenumber *n*, with the amplitude of the sinusoidal wave fixed at $A=20$. The colorbars on the left of (**a**) and (**c**) indicate the colour-scheme adopted for the amplitudes (top) and wavenumbers (bottom) analysed; the black solid lines refer to the reference case of the suspension subject to a plain shear-flow.

## Results

We start by considering a perturbation described by Eq. (1) with $A=20$ and $n=1$. Thus, the particles are driven by a non-uniform shear-rate, due to the sinusoidal disturbance, but under the same mean total shear-rate. The effects of the perturbed shear-rate on the rheology of the suspensions are reported in Fig. 1. In particular, the figure shows the relative viscosity (i.e., the shear-stress ratio) $\overline{\eta }_r=\overline{\sigma }_{12}/\eta _0\overline{\dot{\gamma }}$, where $\overline{\sigma }_{12}$ is the shear component of the suspension stress tensor $\overline{\sigma }$, as a function of the non-dimensional time, i.e. the strain $\gamma =\overline{\dot{\gamma }}t$, for all the possible configurations compatible with the boundary conditions (i.e., the waves $u_{2},u_{3}\sim \sin {(x_1)}$ are discarded since they are not consistent with the Lees-Edwards boundary conditions). In particular, the figure is split into two rows, where the top one sketches the velocity disturbance and the bottom one shows the time-history of $\overline{\eta }_r$, keeping the same colour map for clarity reasons. In the same figure, the black solid lines refers to the reference case, i.e. the particles immersed in a plain shear-flow. Note that, all the cases start from the same initial configuration, that corresponds to a bidisperse suspension with total volume fraction $\overline{\phi }=0.50$, and dispersion ratio $\lambda =a_2/a_1=3$. The suspensions are sheared with a high mean shear-rate, i.e., $\overline{\dot{\gamma }}/\dot{\gamma }_0=1$ (a description of the latter quantity can be found in the Supplementary Material), and such configuration can be located within the shear-thickening region of the flow curve^11,59^. From Fig. 1, we observe that three out of four cases do not show significant alteration in terms of $\overline{\eta }_r$, apart from a small increase of the relative viscosity; on the other hand, the remaining case manifests substantial modifications. This case corresponds to the sinusoidal disturbance acting in the shear plane $x-y$, i.e. $u=20 \overline{\dot{\gamma }} a_0\sin {\left( \kappa _0 y\right) }$ (red solid line) and leads to a reduction of the effective viscosity by approximately $50\%$.

In the following part of this section, we will investigate the cause of the viscosity drop for the latter case mentioned. The investigation will be carried out via a parametric study aiming at unravelling the effect of the amplitude and of the wavenumber of the disturbance.

Figure 2 reports the trends of the relative viscosity obtained varying the amplitude *A* (top row) and the wavenumber $\kappa =n \kappa _0$ (bottom row) of the disturbance. At first, we analyse the effect of the amplitude *A*; in particular, in the top row of Fig. 2, we show the time-history of the relative viscosity $\overline{\eta }_r$ (left panel), and we report the mean values of $\overline{\eta }_r$ collected at statistically steady-state, together with their contributions breakdown (right panel). Starting from the zero-amplitude case, i.e. uniform shear flow (black line), we observe that the effective viscosity monotonically decreases by increasing the amplitude, eventually saturating for $A>20$. The reduction rate is strongly non-linear, with very little changes of $\overline{\eta }_r$ for $A \le 10$, followed by a sudden decrease. This is clearly visible from the mean values of $\overline{\eta }_r$ at statistically steady-state (right panel, total value in the legend). Therefore, as a first result, the present behaviour suggests the existence of a threshold value which activates the mechanism of the reduction of the relative viscosity. Note that, the final value of the effective viscosity is reached after a long transient, i.e. $\gamma \sim 15$, and that increasing the amplitude of the disturbance contributes to shorten the convergence time of the rheological response of the suspension.

Next, we report the trends of the relative viscosity obtained varying the wavenumber $\kappa =n \kappa _0$ of the disturbance. As done for the amplitude *A*, in the bottom row of Fig. 2 we show the time-history of the relative viscosity $\overline{\eta }_r$ (left panel), and we report the mean values of $\overline{\eta }_r$ collected at statistically steady-state, together with their relative contributions (right panel). Similarly to the previous cases, starting from the uniform shear flow (black line), we observe that the effective viscosity non-linearly decreases when a disturbance with higher harmonics is applied to the suspension, reaching a minimum value between $n=4$ and $n=8$. For higher harmonics (e.g., $n=16$), the relative viscosity starts increasing again, thus suggesting the presence of an optimum harmonic that minimizes the value of the relative viscosity of the suspension. This tendency is also confirmed by the mean values of the relative viscosity at statistically steady-state. Another outcome suggested by the time-history of the relative viscosity is that the harmonics with higher wavenumbers significantly reduce the convergence time (as done by the higher amplitudes), nevertheless increasing the overall level of fluctuations, and showing the presence of an optimum value (i.e., faster convergence rate) for wavenumbers included between $n=4$ and $n=8$.

To understand the cause of the viscosity reduction, we give a closer look to the several stress contributions to the effective viscosity. In panel b and d of Fig. 2, we compare the reference case (plain shear) to the suspensions driven by the non-uniform shear flow varying the amplitude *A* of the sinusoidal disturbance (panel b, with $n=1$) and its wavenumber *n* (panel d, with $A=20$). The three components of the stress $\overline{\eta }_r$ are shown: the hydrodynamic contribution which includes the Stokes drag, the lubrication and the Newtonian component of the carrier fluid; the interactions caused by the electrochemical potentials and the stresses due to the particle-particle contacts. The suspensions analysed in the parametric study are located within the shear-thickening region of the flow curve, i.e., $\overline{\dot{\gamma }}/\dot{\gamma }_0=1$, thus, the contributions to the shear-stress related to the contacts and the hydrodynamics are dominant (with the former being more important), while the electrochemical potentials play no effective role. Within the parameters chosen in this work, we observe that the reduction of $\overline{\eta }_r$ (for the cases with $A\ge 20$ and $n\ge 1$) is mostly due to a strong decrease of the contacts contribution to the shear-stress, while the hydrodynamics remains almost unchanged. Worth noticing is the case with $A=20$ and $n=16$, where also the hydrodynamics contribution is significantly reduced compared to the other cases analysed.

**Figure 3:**
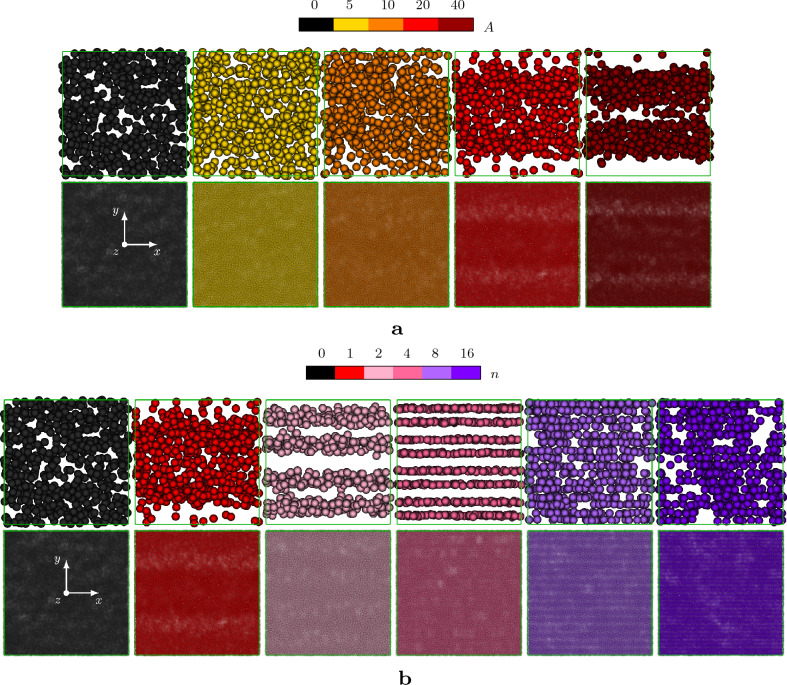
(**a**) Instantaneous snapshots of the suspensions at $\gamma =20$ varying the amplitude of the sinusoidal perturbation, fixing $n=1$. (**b**) Instantaneous snapshots of the suspensions at $\gamma =20$ varying the wavenumber of the sinusoidal perturbation, fixing $A=20$. The top row of the subfigures shows the distribution of the large particles, while the bottom row the respective distribution of the small particles of the suspensions. The colour-scheme reflects the one adopted in the top row of Fig. 2.

To highlight the origin of the relative viscosity reduction, we show in Fig. 3a,b, the instantaneous snapshots of the suspensions studied when a statistically steady-state is reached. In particular, the two panels are organised in a $5\times 2$ (or $6\times 2$) matrix as follows: from left to right, the amplitude (or the wavenumber) increases, from $A=0$ ($n=0$) to $A=40$ ($n=16$); in the top row, the distribution of the larger dispersed phase is shown while, in the bottom row, the location of the smaller dispersed phase is displayed. The two panels qualitatively show the effect of the sinusoidal disturbance on the suspension: the large particles tend to separate from the small ones, forming parallel layers normal to the shearwise (i.e. *y*) direction. The number of layers of large particles seem to be correlated with the wavenumber of the disturbance, while the role of the amplitude appears to be related to a threshold that activates the demixing of the phases, as previously postulated from the mean values of the relative viscosity (i.e., Fig. 2).

**Figure 4:**
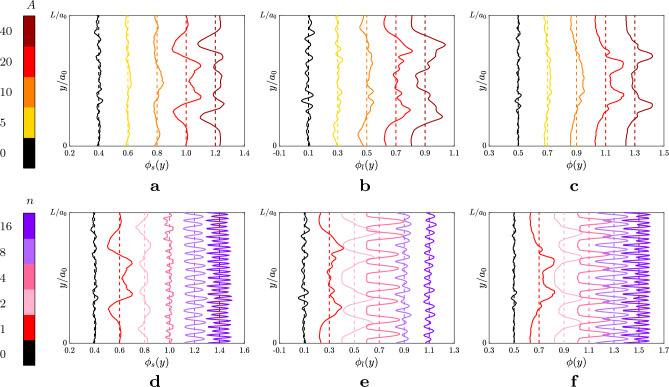
Profiles of the local volume fractions along the shearwise direction *y*. (**a**) and (**d**) show the local volume of the small particles $\phi _s(y)$; (**b**) and (**e**) show the local volume of the large particles $\phi _l(y)$; (**c**) and (**f**) show the total local volume fraction $\phi (y)$. The panels in the top row, i.e. panel **a**–**c**, refer to the sinusoidal disturbance with $n=1$ and increasing amplitude *A*, as shown in the respective legend on the left of the figure; the panels in the bottom row, i.e. (**d**–**f**), refer to the cases with $A=20$ and increasing *n*, as shown in the respective legend on the left of the figure. The black profiles in each panel refer to the reference case with no sinusoidal disturbance. Finally, the dashed lines refer to the values $\overline{\phi }_s=0.4$, $\overline{\phi }_l=0.1$ and $\overline{\phi }=0.5$. Note that, the profiles for different *A* and *n* have been shifted horizontally by 0.2 for visual purpose.

**Figure 5:**
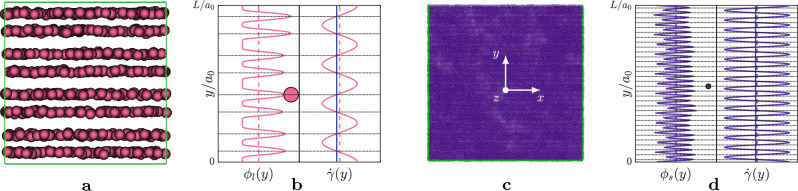
(**a**) and (**c**) instantaneous snapshot of one of the two dispersed phases of the suspension; (**b**) and (**d**) plots of the local volume fraction along the shearwise direction *y* of the respective phase, alongside the local profile of the shear-rate applied to the suspension (right side). (**a**) and (**b**) case $A=20$ and $n=4$, with the snapshot of the distribution of the large phase in the suspension; (**c**) and (**d**) case $A=20$ and $n=16$, with the snapshot of the distribution of the small phase in the suspension. The two plots in (**b**) and (**d**) are split by the vertical, black solid line in the middle of the figure; the other vertical straight lines represent, from left to right: the mean value of the volume fraction of the particles ($\overline{\phi }_l=0.1$ or $\overline{\phi }_s=0.4$, pink/violet dashed line); $\dot{\gamma }(y)=0$ (blue solid line) and the mean value of the total shear-rate applied to the suspension (pink/violet dashed line). The dotted horizontal lines outline the *y* location of $\dot{\gamma }(y)=0$. Finally, the pink/violet circle in the graphs sketches a large/small particle of the suspension.

Quantitatively, this becomes clear when showing the small and large particles concentration along the *y*-coordinate, as pictured in Fig. 4 (note that the symmetry of the profiles is discussed in the Supplementary Material). The figure shows, within each row, the volume fraction of the small particles $\phi _s(y)$, that of the large particles $\phi _l(y)$ and the total volume fraction $\phi (y)$, as a function of the amplitude of the disturbance with wavenumber $n=1$ (top row), and as a function of the wavenumber *n*, fixing the amplitude to $A=20$ (bottom row). Note that the local volume fractions oscillate around the values $\overline{\phi }_s = 0.4$, $\overline{\phi }_l=0.1$ and $\overline{\phi }=0.5$, highlighted by the dashed lines; therefore, the labels on the abscissas simply indicate the aforementioned values shifted by an offset to show all the curves on a single graph. From the top row of Fig. 4, we observe the progressive formation of two regions with high large particles concentration $\overline{\phi }_l$, with the peaks expanding monotonically by increasing the amplitude of the disturbance. On the other hand, in the bottom row of Fig. 4, as *n* grows we observe an increase of the number of layers where the large particles are collected, thus suggesting that the number of accumulation regions is controlled by the wavelength of the disturbance with the amplitude controlling only the amount of the relative concentration. This quantitative analysis corroborates what observed in the snapshots shown in Fig. 3. Panel d and e of Fig. 4 also show that, for small wavenumbers, i.e., $n\le 8$, the large particles accumulate into well-separated layers while, for large wavenumbers, i.e., $n>8$, the small ones do. This is a consequence of the size of the particles compared to the wavelength of the perturbation: in the former case, the large particles have a diameter comparable to or smaller than the wavelength of the sinusoidal wave, defined as $\Lambda =1/(n\kappa _0)$, while in the latter case, the small ones do. Indeed, when the wavelength of the disturbance is much smaller than the particle size, its effect is filtered out by the particle size, with the particles feeling the disturbance as noise in the limit of $\Lambda \ll a_1,a_2$. This becomes clear when looking at the local distributions of the large particles for $n=8$ and $n=16$ in panel e of Fig. 4: the two curves show the same number of peaks (16), meaning that the effect of the wavelength on the large particles had reached a maximum and saturated. An estimate of the maximum value of the wavenumber felt by particles of radius $a_i$ (with $i=1,2$) can be easily computed as

6 $$\begin{aligned} n_{max} \approx \dfrac{1}{2}\dfrac{L}{2 a_i}, \end{aligned}$$

which for the large particles we consider gives $n_{max} \approx 7$, comparable to $n=8$. The wavenumber $n_{max}$ is half the ratio between *L* and *d* because of the points of accumulation of the particles, which are located in the proximity of the zero-shear values, as it can be seen in Fig. 5. In particular, panel a of the figure shows a snapshot at statistically steady-state of the large particles for the case $A=20$ and $n=4$, while in panel b the mean local volume fraction of the large particles (left side) together with the shape of the local shear-rate (right side) are plotted. From panel a and b, we can see how the large particles accumulate in parallel layers around the locations of the zero-shear regions (dotted horizontal lines in panel b). On the other hand, when considering disturbances with high wavenumbers, the smaller particles are reorganizing in parallel layers, as can be observed in panel c and d of Fig. 5. The two panels mirror panel a and b for the case $A=20$ and $n=16$. For this case, it is clear that the small particles collect around the zero-shear regions.

**Figure 6:**
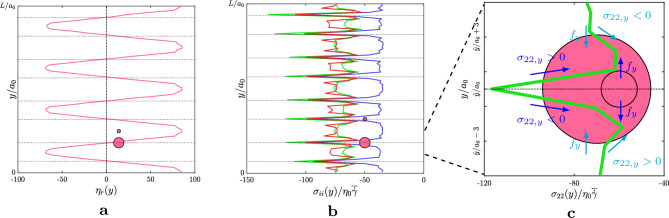
(**a**) Local distribution of the mean shear-stress ratio $\eta _r(y)$ along the shearwise direction *y*. The vertical dotted line highlights $\eta _r(y)=0$; the two circles sketch the two types of particles dispersed in the suspension. (**b**) Local distribution of the mean normal stresses, $\sigma _{11}(y)$ (red line), $\sigma _{22}(y)$ (green line) and $\sigma _{33}(y)$ (blue line), along the shearwise direction *y*. (**c**) Zoom of $\sigma _{22}(y)$ in one of the regions of (**b**) bounded by the dashed lines. The location $\hat{y}/a_0$ corresponds to the zero-shear location $\dot{\gamma }(y)=0$. The two circles represent the particles of the two phases of the suspension. The arrows show the directions of the gradients of the normal stress $\sigma _{22,y}$ and the respective forces they generate on the particles. In all the panels of the figure, the horizontal dotted lines indicate the regions at $\dot{\gamma }(y)=0$. The case considered has a sinusoidal perturbation with $A=20$ and $n=4$.

Next, to understand the mechanism of the particles accumulation, in Fig. 6 we show the relevant components of the local relative mean stress tensor for the case $A=20$ and $n=4$ at statistically steady-state. In particular, panel a and b show the local relative viscosity (i.e., the local shear-stress ratio) and the local relative normal components of the stress tensor, respectively, along the shearwise direction *y*. Note that the integral value of the local shear-stress ratio along the shear-wise direction *y* corresponds to the mean relative viscosity. In these panels, the dotted horizontal lines outline the zeros of the total shear-rate profile, i.e., $\dot{\gamma }(y)=0$. The profile of the local shear-stress ratio promptly highlights that the suspension is sheared in two opposite directions (opposite signs of $\eta _r(y)$) following the shape of the imposed shear-rate, with regions of zero shear-stress in the proximity of the large particles collection points (i.e., $\dot{\gamma }(y)=0$). Focusing on the normal stresses, instead, it is clear that, in the regions where the large particles accumulate, there are peaks of high-compressive stresses along the three directions. For $\sigma _{11}(y)$ (red line) and $\sigma _{22}(y)$ (green line) those peaks are enclosed by two adjacent local maxima, whose distance is comparable with the diameter of the large particles. A zoom on one of the regions enclosing the three aforementioned peaks (one minimum and two maxima) is shown in panel c of Fig. 6. In particular, only the normal stress along the shearwise direction $\sigma _{22}(y)$ is reported. Furthermore, in the figure we also sketch the force $f_y$ that the gradients of the normal stress $\sigma _{22,y}$ generate on the particles: we notice how the gradients enclosing the minimum peak tend to push away the particles from the zero-shear regions, while the two local maxima have an opposite effect. From the sketch, it is clear that small particles are effectively pushed out of the zero-shear regions, while the large particles are trapped by the two small stabilising peaks of $\sigma _{22}(y)$. Indeed, the effect of the stronger peak in the proximity of the zero-shear regions is filtered out by the large particles due to its size. This explains the mechanism of the particles migration for this configuration: the small particles migrate away from the zero-shear regions and, in turn, push the large particles in the accumulation points, eventually locking them there by acting with compressive forces on the extrema of the large particles.

**Figure 7:**
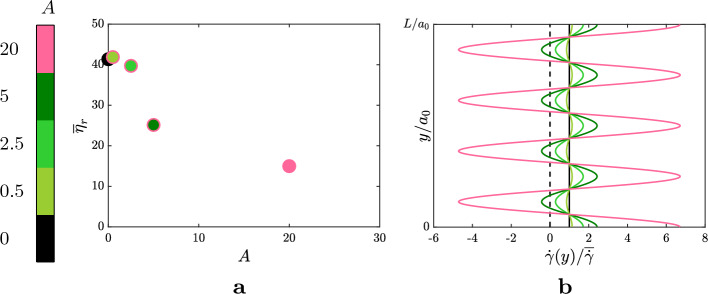
Wavenumber $n=4$. (**a**) Mean relative viscosity $\eta _r$ at statistically steady-state for the disturbance $u=A \overline{\dot{\gamma }} a_0 \sin {\left( 4 \kappa _0 y \right) }$, varying the amplitude *A* of the disturbance. (**b**) Profiles of the shear-rate applied to the suspensions along the shearwise direction *y*. The colour-scheme indicates the amplitude imposed and reflects the one used in the colorbar. The black dashed line is the zero shear-rate.

One of the reasons that trigger the phase separation and the demixing process can be sought in the strength of the forcing term that imposes positive and negative shear-rates to the suspension. To investigate this, we carry out a further parametric study where we choose the case with the streamwise wave having $n=4$ (that showed an efficient demixing) and we vary the amplitude of the wavy disturbance. Panel a of Fig. 7 reports the values of the relative viscosity collected at statistically steady-state for the cases with amplitude $A=[0.5,2.5,5,20]$, compared to the reference case, i.e. the suspension subject to a plain shear flow (black marker with $A=0$). The figure shows a similar non-linear trend seen for the cases with $n=1$, with a strong reduction of the relative viscosity between $A=2.5$ and $A=5$. In panel b, instead, we report the profiles of the applied shear-rates along *y*. Note that the case with $A=5$ is the first one to cross the zero-shear line (dashed black line). This suggests that, when the disturbance is high enough to produce negative shear-rates, the demixing is activated causing a sudden reduction of $\overline{\eta }_r$. Note that, being the total shear-rate $\dot{\gamma }(y)=\overline{\dot{\gamma }} + A \overline{\dot{\gamma }} a_0 n \kappa _0 \cos {(n \kappa _0 y)}$, either a disturbance with a strong amplitude or one with a large wavenumber (or a combination of the two) may cause the demixing of the dispersed phases.

**Figure 8:**
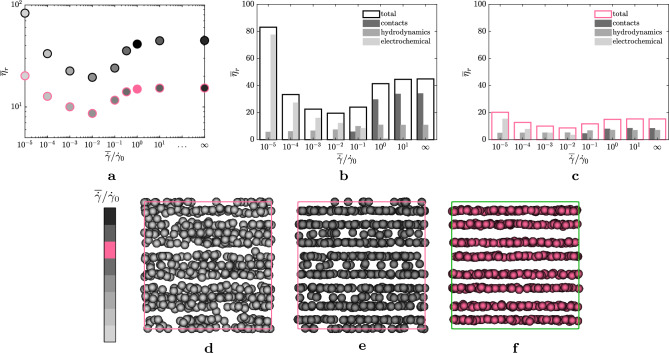
Top row, (**a**–**c**). (**a**) Mean relative viscosity $\eta _r$ at statistically steady-state for suspensions driven by a plain shear-flow (markers with a black edge) and for the suspension subject to the disturbance $u=20 \overline{\dot{\gamma }} a_0 \sin {\left( 4 \kappa _0 y \right) }$ (markers with a pink edge) as a function of the shear-rate $\overline{\dot{\gamma }}/\dot{\gamma }_0$. (**b**) and (**c**) Contributions to the mean relative viscosity $\overline{\eta }_r$ accumulated at statistically-steady state of the contacts, hydrodynamics and electrochemical potentials (the respective colours are shown in the legend within the panels), as a function of the shear-rate $\overline{\dot{\gamma }}/\dot{\gamma }_0$, for the suspension driven by a plain shear-flow (**b**) and for the suspension subject to the sinusoidal disturbance ( **c**). Bottom row, (**d**–**f**) instantaneous snapshots of the large particles of the suspensions at statistically-steady state varying $\overline{\dot{\gamma }}/\dot{\gamma }_0$. (**d**) $\overline{\dot{\gamma }}/\dot{\gamma }_0=10^{-5}$; (**e**) $\overline{\dot{\gamma }}/\dot{\gamma }_0=10^{-2}$; (**f**) $\overline{\dot{\gamma }}/\dot{\gamma }_0=10^{0}$. The colours in (**a**) and (**d**–**f**) refer to the colormap in the figure. Note that the colour pink can be changed to black in (**a**) to show the non-perturbed case. The suspensions reported are subject to the sinusoidal perturbation with $A=20$ and $n=4$.

The final question of this work concerns the extension of the demixing process seen within the manuscript for the other rheological regimes of the dense suspensions, i.e. for any point belonging to the whole flow-curve ($\overline{\eta }_r$ as a function of the shear-rate). To introduce the shear-rate dependency, we tune the amplitude of the electrochemical as in Eq. (5) (see also^11^). To determine the Hamaker constant and the intensity of the repulsive forces separately, we set the ratio $|\varvec{F}_R |/ |\varvec{F}_A |= 9$ when two particles are at contact $d_{ij}=0$. Panel a of Fig. 8 shows the values of the relative viscosity collected at statistically steady-state for the suspensions immersed in a plain shear flow (markers with the black edge) and for the suspensions subject to the sinusoidal disturbance with $A=20$ and $n=4$ (markers with the pink edge), varying the shear-rate $\overline{\dot{\gamma }}/\dot{\gamma }_0$. Panel b and c, instead, show the decomposition of the relative viscosity in the three main contributions (as seen in Fig. 2 already) for the suspension subject to the plain shear-flow and the sinusoidal shear-flow, respectively. As it can be seen, the relative viscosity is always lower when a sinusoidal shear is imposed to the suspension for any $\overline{\dot{\gamma }}/\dot{\gamma }_0$, meaning that the demixing is always activated, despite the different nature of the contributions that govern the rheology of the suspensions in the various regimes, as it can be seen from the histograms. In particular, while for high $\overline{\dot{\gamma }}/\dot{\gamma }_0$ the reduction of the relative viscosity is due to a reduction of the contacts, as discussed before, for low $\overline{\dot{\gamma }}/\dot{\gamma }_0$ this is due to a reduction of the electrochemical interactions. This suggests that the segregation phenomenon in this case is driven by the response of the suspension to the high shear introduced by the sinusoidal wave, that in turns activates the selective process carried out by the normal stresses. The effect of the different contributions controlling the particle dynamics in the various regimes, however, can be spotted from the instantaneous snapshots of the large particles at statistically-steady state, as shown in the bottom row, i.e. panels d–f, of Fig. 8. In particular, panel d–f show the instantaneous distributions of the large particles for $\overline{\dot{\gamma }}/\dot{\gamma }_0=10^{-5}$, $\overline{\dot{\gamma }}/\dot{\gamma }_0=10^{-2}$ and $\overline{\dot{\gamma }}/\dot{\gamma }_0=10^{0}$, respectively. These three particular scenarios have been selected based on panel c of Fig. 8 to show the effect of the different contributions: in fact, in the configuration of panel d, the dominant contribution is given by the electrochemical potentials (i.e., forces interactions along the centre-to-centre direction); in that of panel e, the dominant contribution is given by the hydrodynamics, while in the condition of panel f by the normal and frictional contacts. From the figure, it can be seen that when the contributions to the stress tensor are dominated by forces along the centre-to-centre direction (such as in panel d), the large particles accumulate in the region with positive shear-rate (see the pink profile in panel b of Fig. 7) while, when forces with tangential direction start appearing (gradually increasing from panel e to panel f), the large particles tend to stabilise around the zero-shear regions only.

## Discussion

We have performed numerical simulations of dense binary suspensions with a large dispersion ratio, under a non-uniform shear flow, consisting of the combination of a linear shear and a sinusoidal disturbance. First, we find that the only disturbance altering the rheology of the suspension is the one acting on the streamwise component of the velocity in the shear-plane. By varying the amplitude and wavenumber of the perturbation, we discover that the demixing process can be triggered when the local shear-rate becomes negative. When the phases separate, the relative viscosity reduces in the whole flow curve. We explain the process by analysing the full stress tensor of the suspension: we find that large particles are locked in the accumulation points by compressive forces, while the small ones are pushed out by the gradients of the normal stresses. With this analysis, we highlight the importance of the full stress tensor to unravel the complex rhelogical behaviour of dense suspensions.

The phenomenon of particle separation was examined throughout the entire rheological curve under optimal conditions ($A=20$ and $n=4$). Regardless of the shear rate applied to the suspension, the suspension undergoes phase-separation, although the relative significance of the factors contributing to shear stress have a different nature. On the right side of the flow curve, contact interactions dominate, while in the shear-thinning region, electrochemical potentials take precedence. This demixing effect leads to the accumulation of larger particles (for $n \le 4$ in our study) in different regions along the direction of shear: the zeros of the shear-rate for conctact-dominated suspesions, positive shear-rate for electochemical potentials-dominated interactions.

It is important to underline that, to properly understand the demixing, considering the finite size of the particles is essential to filter out and select the proper information from the gradients of the normal stress tensor. Therefore, caution should be used when employing point-size particles models to explain or analyse such phenomena.

The outcomes of our analysis have far-reaching implications within several scientific disciplines, specifically rheology, microfluidics, biofluidics, and granular flows. By delving into these areas, our research contributes to a deeper understanding of the behaviour of various materials and fluids under different conditions.

In the field of rheology, our findings provide a foundational basis for developing a comprehensive rheological model that accurately captures the complexities of real-world situations. Traditionally, rheological models have overlooked structural inhomogeneity or natural disturbances, which can have substantial effects on material behaviour. However, our study addresses this limitation by considering these factors, thus offering a more realistic depiction of rheological phenomena. Moreover, our research extends its impact to the realm of microfluidics, where the manipulation and control of fluids at the microscale are essential. By incorporating our insights into microfluidic systems, researchers and engineers can gain a better understanding of how spatial disturbances influence the fluid flow and behaviour, enabling them to design more efficient and precise microfluidic devices. Additionally, our research contributes to the study of granular flows. By considering spatial disturbances and their effects on granular materials, we offer a more comprehensive framework for modelling and analysing granular flow behaviour, thereby facilitating improved process design and optimisation.

An exciting future direction for our study involves exploring the coupling of spatial disturbances with the more commonly studied temporal disturbances. By incorporating both spatial and temporal factors into our model, we can take a significant stride towards simulating and understanding real-world conditions more accurately. This advancement would enable researchers to investigate how disturbances propagate and interact over time, providing a more holistic understanding of complex systems and further bridging the gap between theoretical models and real-world scenarios.

### Supplementary Information

Supplementary Information.

## Data Availability

The code used for the present research is a fully validated software, *CFF-Ball-0x*, publicly available at https://github.com/marco-rosti/CFF-Ball-0x. All the reported results can be reproduced using this code and the information provided in the text. All data are available from the corresponding author upon reasonable request.
