# Supplementary material for: Dense bidisperse suspensions under non-homogeneous shear
Source: Sci Rep. 2023 Aug 31;13:14310. doi: 10.1038/s41598-023-41587-3 (PMC10471770; doi:10.1038/s41598-023-41587-3)
Supplement: Supplementary file 1 — Supplementary Information. [file 41598_2023_41587_MOESM1_ESM.pdf]

Supplementary information to: Dense bidisperse suspensions under non-homogeneous shear

Alessandro Monti* (alessandro.monti@oist.jp) and Marco Edoardo Rosti* (marco.rosti@oist.jp)
Complex Fluids and Flows Unit, Okinawa Institute of Science and Technology Graduate University, 1919-1 Tancha, Onna-son, Okinawa 904-0495, Japan
(Dated: August 18, 2023)

SUPPLEMENTARY MATERIAL TO: METHODS

In this appendix, we report the expressions of the forces acting on the particles considered in this study, and provide more details on the numerical method. In particular, in our model we consider the hydrodynamics, inelastic contacts and electrochemical potentials, and indicate them by the superscripts H, C and E,respectively.

Concerning the hydrodynamics, dense suspensions of rigid particles immersed in a low-Reynolds-number flow are subject to a Stokes drag and a pair-wise, short-range lubrication force [1] caused by the reaction of the fluid squeezed in the narrow gaps between two neighbouring particles. This contribution is modelled in our software as a linear relationship between the forces and the velocities, $\boldsymbol{F}^H = -\mathbb{R}(\boldsymbol{u}-\boldsymbol{U}^\infty)$, where $\mathbb{R}$ is a resistance matrix that is obtained by neglecting the far-field effects and considering only the dominant near-field (threshold gap between particles $\delta_{lub} < a_0$) divergent elements coming from the squeeze, shear and pump modes [1, 2]. More details on the coefficients of the resistance matrix adopted can be found in Monti *et al.* [3].

The contacts, instead, are replicated with the stick-and-slide method [4], that mimics inelastic, frictional contacts between a pair of slightly overlapping particles with spring-dashpot systems placed in the normal (centre-to-centre) and tangential directions. Considering two particles, i and j, the stick-and-slide model can be written as

$$\boldsymbol{F}^C_{n,ij} = k_n \delta_{ij} \boldsymbol{n} + \gamma_n \boldsymbol{u}_{n,ij}, \tag{1a}$$

$$\boldsymbol{F}^C_{t,ij} = k_t \xi_{ij} \boldsymbol{t}, \tag{1b}$$

$$\boldsymbol{T}^C_{ij} = a_i \boldsymbol{n} \times \boldsymbol{F}^C_{t,ij}, \tag{1c}$$

where the vectors $\boldsymbol{n}$ and $\boldsymbol{t}$, together with the subscripts n and t, indicate the directions of the force (i.e. normal and tangential) and the parameters k_n, k_t and γ_n are the spring constants and the normal damping constant, respectively. The velocity $\boldsymbol{u}_{n,ij}$ is the projection of the relative velocity vector between the particles ith and jth projected in the normal direction, while δ_{ij} and ξ_{ij} represents the displacement of the normal and tangential spring. The latter is an integral length that considers the history of the contact [4] and its value is such that the tangential force satisfies the Coulomb's law $|\boldsymbol{F}^C_{t,ij}| \leq \mu_C |\boldsymbol{F}^C_{n,ij}|$, where μ_C is the friction coefficient (in this work, $\mu_C = 0.5$ for all the suspensions simulated).

Finally, the contribution from the electrochemical potentials is modelled as a combination of a distance-decaying repulsive force (with the distance-decay length scale set by the screening length) and an attractive force expressed in the van der Waals form [5, 6],

$$\boldsymbol{F}^R_{ij} = -F_0 e^{-d_{ij}/L_S}\, \boldsymbol{n}, \qquad \text{if } d_{ij} \geq 0, \tag{2a}$$

$$\boldsymbol{F}^A_{ij} = \frac{H_A \overline{a}}{12(d^2_{ij} + \varepsilon^2)} \boldsymbol{n} \qquad \text{if } d_{ij} \geq 0. \tag{2b}$$

In Equation (2a), F_0 is the amplitude of the repulsive force, $\overline{a} = 2a_i a_j/(a_i + a_j)$ the harmonic radius and L_S is the screening length that controls the decay of the force. In Equation (2b), instead, H_A is the Hamaker constant and ε is a small regularisation term (generally $\epsilon \approx 0.01\overline{a}$) introduced to avoid the singularity at contact, when the surface distance between the particles $d_{ij} = 0$.

All these contributions induce mechanical stress in the suspension and the stress tensor σ can be evaluated through the stresslet theory [7]. From σ, all the rheological quantities (relative viscosity η_r, first and second normal stress differences N_1 and N_2 and pressure Π) can be extrapolated.

The physics of the suspension described by the Newton-Euler equations is dominated by three non-dimensional groups obtained by applying the Buckingham Pi theorem and choosing as fundamental quantities the properties that describe the hydrodynamic force, i.e. $F^H \propto \eta_0 a_0^2 \bar{\dot{\gamma}}$. The non-dimensional groups are listed in the main manuscript.
In equation (4) of the manuscript, k_n approximates the equivalent stiffness of the spring-dashpot systems,

$$k_{eq} = k_n \left(1 + \frac{k_t}{k_n} + \frac{\gamma_n \bar{\dot{\gamma}}}{k_n}\right), \tag{3}$$

with the additional constraints $k_t \ll k_n$ and $\gamma_n \bar{\dot{\gamma}}/k_n \ll 1$ [1]. In equation (5) of the manuscript, we defined a new shear-rate, $\dot{\gamma}_0 = F^E/\eta_0 a_0^2$, being $F^E = |\boldsymbol{F}^E(d_{ij} = 0)| = F^R + F^A$ (with $F^R = |\boldsymbol{F}^R_{ij}(d_{ij} = 0)|$ and $F^A = |\boldsymbol{F}^A_{ij}(d_{ij} = 0)|$). in F^E, the dominant contribution is given by the repulsive forces, with the choice $F^R = 0.9F^E$ and $F^A = 0.1F^E$. This non-dimensional group represents the additional time-scale introduced by the electrochemical contribution and it can be thought of as the equivalent of the Péclet number for the Brownian-suspensions;

thus, tuning the module of the repulsive force F^R and the Hamaker constant appropriately, a shear-rate dependency can be imposed to the suspension. Note that in the manuscript, at times we omitted the subscript ij for the sake of readability.

From a computational viewpoint, the governing equations (2) in the manuscript, are discretized and advanced in time with the modified velocity-Verlet explicit scheme [8]. The modified velocity-Verlet scheme is second-order accurate in time and, being explicit, it requires a time-step Δt able to capture the shortest dynamics of the systems, typically established by the stiffness of the contacts; a good indicator of the time-step required can be obtained by building the time constant from the normal spring-dashpot system, i.e. $\Delta t\bar{\dot{\gamma}} = \gamma_n\bar{\dot{\gamma}}/k_n$. Finally, as already mentioned above, the computational domain is a cubic box of size L^3. The shear-rate $\bar{\dot{\gamma}}$ is applied along the y direction, with the Lees-Edwards boundary conditions [9] preserving the ideality of the flow, removing the effect of the walls.

The set of parameters for carrying out these simulations are listed in Table I. Note that, for the sake of simplicity and completeness, $a_0 = a_1 = 1\ \mathcal{L}$, $\bar{\dot{\gamma}} = 1\ \mathcal{T}^{-1}$ and $\rho_p = 1\ \mathcal{M}\,\mathcal{L}^{-3}$. Here $\mathcal{L}$, $\mathcal{T}$ and $\mathcal{M}$ are the typical scales for length, time and mass, respectively. From Table I, all the parameters used in the simulations can be retrieved:

$$
\begin{aligned}
\eta_0 &= \frac{\rho_p a_0^2 \bar{\dot{\gamma}}}{St};\\
k_n &= \hat{k}\eta_0 a_0 \bar{\dot{\gamma}};\\
k_t &= \frac{2}{7}k_n;\\
\gamma_n &= 10^{-7}\frac{k_n}{\bar{\dot{\gamma}}};\\
F^E &= (6\pi)\eta_0 a_0^2 \dot{\gamma}_0;\\
F^A &= \frac{F^E}{10} \quad \text{therefore, Hamaker constant;}\\
F^R &= 9F^A \quad \text{therefore, } F_0.
\end{aligned}
$$

Note that, for F^E we multiply the non-dimensional group for the value 6π, that is the value that premultiplies the viscous force in the Stokes drag of a sphere [see 1, where the Stokes drag of a sphere has been chosen as reference value].

SUPPLEMENTARY MATERIAL TO: RESULTS

Symmetry of the local volume fraction profiles

Concerning the possible lack of symmetry of the profiles in Figure 4 of the main manuscript, we prove here that the lack of averaging and the finite number of particles (only 600 large particles) are to blame. In particular, we considered three initial conditions to the 8 cases with the sinusoidal perturbation, obtained by sampling the simulation with no perturbation (i.e. $A = 0$ and $n = 0$) at different time instants. The cases with identical amplitude and wavenumber are then ensemble averaged, together with 900 instantaneous fields per run sampled after convergence. Figures 1 and 2 compare the profiles with and without ensemble average respectively. The improvement of the symmetry is clearly visible. Therefore, the lack of perfect symmetry in the volume faction profiles is related to the lack of averaging only, together with the finite number of particles.

Finally, we also prove the correctness of our implementation of the Lees-Edwards boundary conditions. We selected the run with $A = 20$ and $n = 1$ (red curve in Figures 1 and 2) and we averaged its volume fraction distributions with an identical set-up whose streamwise velocity profile is shifted of a value $u = 30a_0\bar{\dot{\gamma}}$. In case of a wrong implementation of the boundary conditions, the resulting local volume fraction should differ, while the red profiles in Figure 2 demonstrate the correctness of the implementation.

[1] R. Mari, R. Seto, J. Morris, and M. Denn, Journal of Rheology **58**, 1693 (2014).
[2] R. Ball and J. Melrose, Physica A: Statistical Mechanics and its Applications **247**, 444 (1997).
[3] A. Monti, V. Rathee, A. Q. Shen, and M. E. Rosti, Physics of Fluids **33**, 103314 (2021).
[4] S. Luding, Granular Matter **10**, 235 (2008).
[5] L. Gálvez, S. de Beer, D. van der Meer, and A. Pons, Physical Review E **95**, 030602 (2017).
[6] A. Singh, S. Pednekar, J. Chun, M. Denn, and J. Morris, Physical Review Letters **122**, 098004 (2019).
[7] E. Guazzelli and J. Morris, *A physical introduction to suspension dynamics*, Vol. 45 (Cambridge University Press, 2011).
[8] R. Groot and P. Warren, The Journal of Chemical Physics **107**, 4423 (1997).
[9] A. Lees and S. Edwards, Journal of Physics C: Solid State Physics **5**, 1921 (1972).

ϕ	N	a_2/a_1	V_2/V_1	St	$\hat{k}$	k_t/k_n	$\gamma_n\bar{\dot{\gamma}}/k_n$	μ_C	$\bar{\dot{\gamma}}/\dot{\gamma}_0$	δ_{lub}/a_0	L_S/a_0	F^R/F^A
0.50	2^{16}	3	0.25	10^{-3}	4×10^5	2/7	10^{-7}	0.5	$\{10^{-5},\ldots,\infty\}$	0.25	0.02	9

TABLE I: Set of parameters used for simulating the behaviour of dense suspensions varying the shear rate. Here, $a_0=a_1$ is the reference length scale.

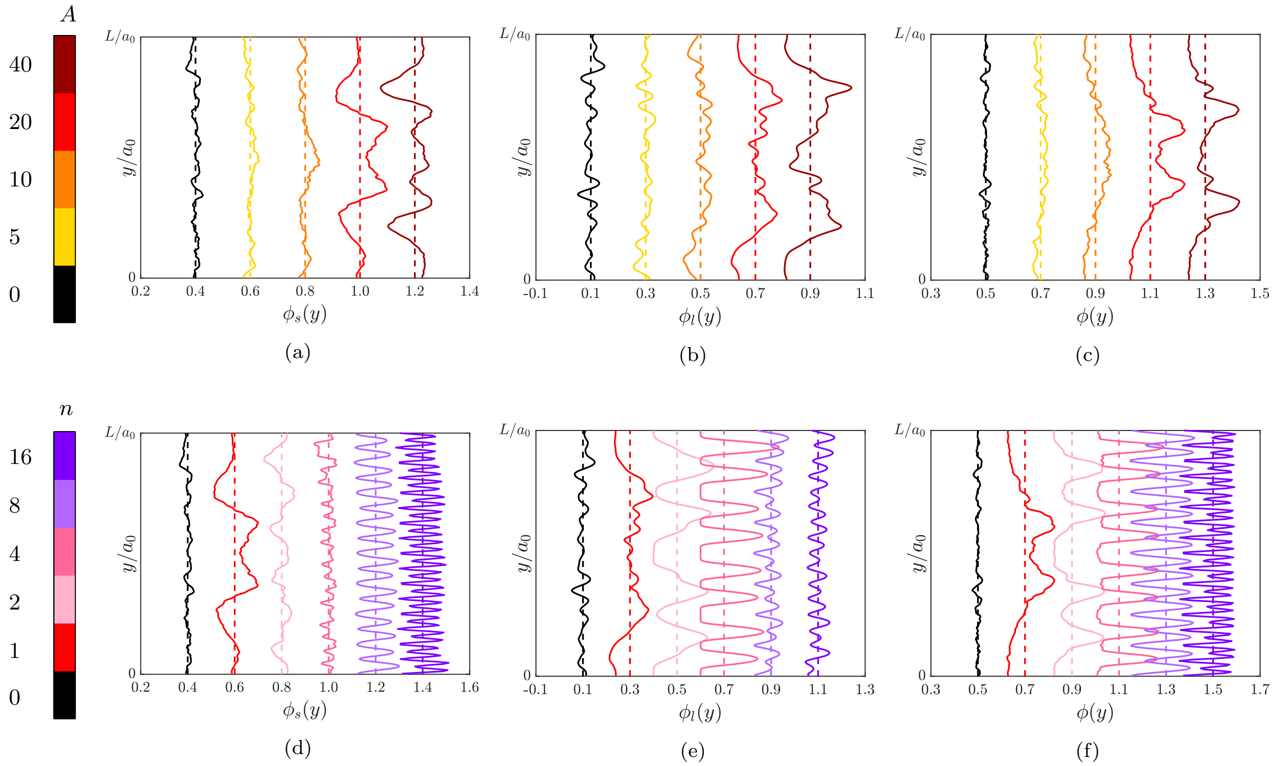

FIG. 1: Profiles of the local volume fractions along the shearwise direction y. Panel **a** and **d** show the local volume of the small particles $\phi_s(y)$; panel **b** and **e** show the local volume of the large particles $\phi_l(y)$; panel **c** and **f** show the total local volume fraction $\phi(y)$. The panels in the top row, i.e. panel **a**–**c**, refer to the sinusoidal disturbance with $n=1$ and increasing amplitude A, as shown in the respective legend on the left of the figure; the panels in the bottom row, i.e. panel **d**–**f**, refer to the cases with $A=20$ and increasing n, as shown in the respective legend on the left of the figure. The black profiles in each panel refer to the reference case with no sinusoidal disturbance. Finally, the dashed lines refer to the values $\overline{\phi}_s=0.4$, $\overline{\phi}_l=0.1$ and $\overline{\phi}=0.5$. Note that, the profiles for different A and n have been shifted horizontally by 0.2 for visual purpose. Version with no ensemble average.

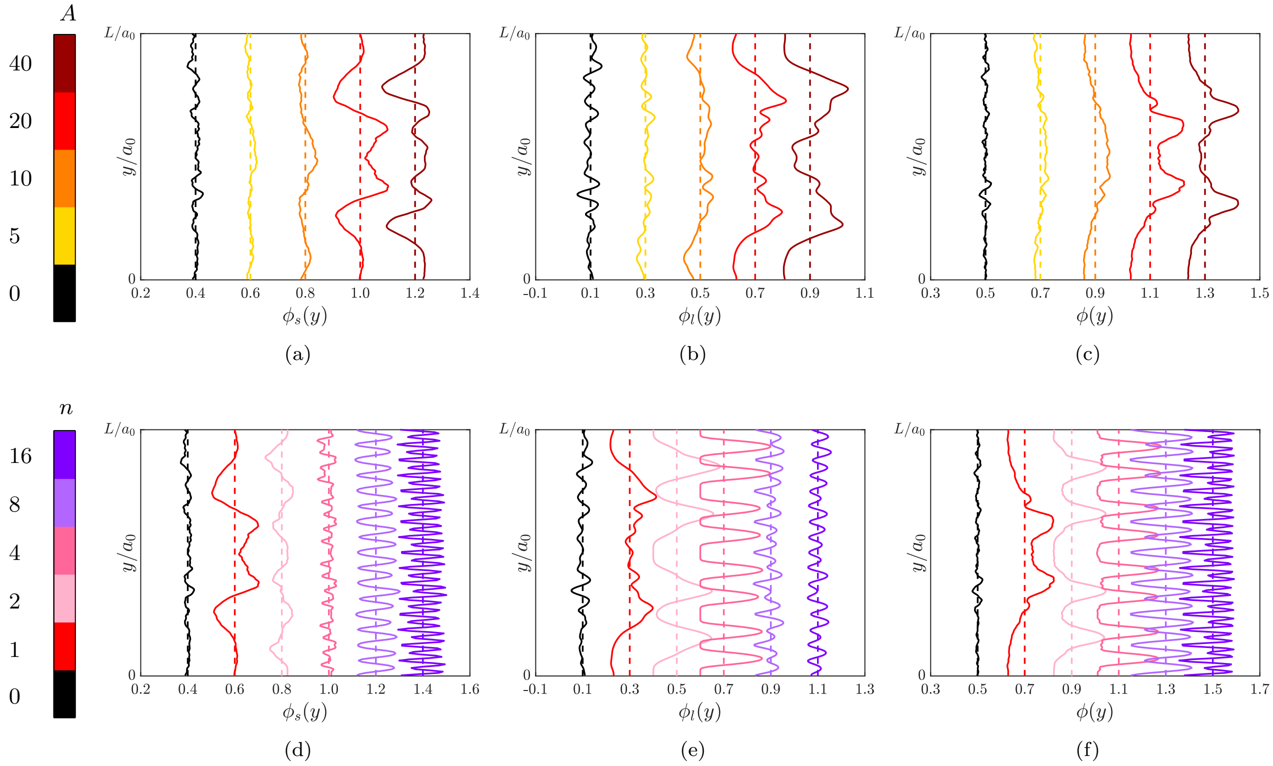

FIG. 2: Profiles of the local volume fractions along the shearwise direction y. Panel **a** and **d** show the local volume of the small particles $\phi_s(y)$; panel **b** and **e** show the local volume of the large particles $\phi_l(y)$; panel **c** and **f** show the total local volume fraction $\phi(y)$. The panels in the top row, i.e. panel **a**–**c**, refer to the sinusoidal disturbance with $n = 1$ and increasing amplitude A, as shown in the respective legend on the left of the figure; the panels in the bottom row, i.e. panel **d**–**f**, refer to the cases with $A = 20$ and increasing n, as shown in the respective legend on the left of the figure. The black profiles in each panel refer to the reference case with no sinusoidal disturbance. Finally, the dashed lines refer to the values $\overline{\phi}_s = 0.4$, $\overline{\phi}_l = 0.1$ and $\overline{\phi} = 0.5$. Note that, the profiles for different A and n have been shifted horizontally by 0.2 for visual purpose. Version with ensemble average.
